# An efficient method for protoplast-mediated production of transformed castor bean (*Ricinus communis*) lines

**DOI:** 10.1186/s13104-023-06414-y

**Published:** 2023-07-06

**Authors:** Paula Figueroa-Varela, Danna Susunaga-Gómez, Catalina Restrepo-Osorio, Carsten Harms, Diego Villanueva-Mejía

**Affiliations:** 1 CIBIOP Research Group, School of Applied Sciences and Engineering, EAFIT University, Medellín, Colombia; 2 Bremerhaven Institute for Applied Molecular Biology, University of Applied Sciences Bremerhaven, Bremerhaven, Germany

**Keywords:** Protoplasts, Cellulase, Macerozyme, PEG-mediated transfection

## Abstract

**Objective:**

The purpose of this study was to develop a method for the isolation, culture, and PEG-mediated protoplast transfection from leaves of in vitro*-*grown plants of *Ricinus communis*.

**Results:**

Factors such as the enzymatic composition and the incubation time were evaluated. The enzymatic solution, containing 1.6% Cellulase-R10 and 0.8% Macerozyme-R10, with 16 h of incubation, was the best condition to achieve a high protoplast yield (481.16 × 10^4^ protoplasts/g FW) with a high percentage of viability (95%). The combination and concentration of enzymes have been shown to affect the protoplast isolation efficiency significantly. Furthermore, we found that a higher number of protoplasts (8.5 × 10^5^ protoplast/g FW) was obtained at a longer incubation time, but their viability decreased. We obtained a simple and efficient protocol to isolate protoplast from *Ricinus communis* leaves and culture. A PEG-mediated protoplast transfection protocol was also established to introduce plasmid DNA into *Ricinus communis* genotypes cultivated in Colombia. Thus, strengthening advances in the genetic improvement processes for this crop are presented.

**Supplementary Information:**

The online version contains supplementary material available at 10.1186/s13104-023-06414-y.

## Introduction

*Ricinus communis* L., known as castor bean, is an essential non-edible oil plant belonging to the Euphorbiaceae family [1], that is considered an industrial raw material due to its oil [2, 3], high level of ricinoleic acid and its wide variety of applications [3, 4]. Castor oil is used in the preparation of brake fluids; as an ingredient for soaps, lubricants, inks, paint, and varnishes; and as the main ingredient in motor oils for high-speed automobile engines [3]. Efficient and replicable de novo plant regeneration methods are crucial to developing biotech-based applications [5], supplying a large-scale regeneration of plants using single selected cells, e.g., protoplast with non-chimeric characters. Several authors reported through indirect organogenesis successfully applied in the Euphorbiaceae family [6–9]; nevertheless, castor bean has high in vitro recalcitrance -the incapacity of plants cells and tissues to respond to in vitro tissue culture [10]- and a few cases of successful regeneration of adventitious shoots have been reported [4, 11–13].

Protoplasts refer to plant cells that have had their cell walls removed, also known as naked cells [14]. Protocols on protoplast isolation and purification, using mechanical and enzymatic methods, have been published to optimize production and reproducibility. Mechanical methods are rarely employed for protoplast isolation [15], since in some cases, the forces necessary to rupture the cell wall damage the more fragile organelles [16]. On the other hand, the effectiveness of enzymatic methods is limited to a few plant species [17]. These restrictions are influenced by factors such as the composition and thickness of the cell wall, temperature, enzyme incubation, pH, agitation, and osmotic solution [18]. Once isolated, protoplasts are remarkable material for plant breeding programs [19, 20], producing new cultivars with desirable traits [21, 22]. Protoplasts have several applications; among the most common is their use as a material to carry out genetic modifications, because it allows increasing the transformation efficiency using foreign DNA [23]; cell fusion allows the generation of somatic hybrids in species with sexual incompatibility [24] and production of metabolites, which are easily released by the protoplasts into the culture medium, avoiding the restrictions of the cell wall [25–27]. For these applications, high concentrations of protoplasts with a high percentage of viability are needed [25]. Recently, protoplasts have been used in genome editing applications, in vivo assays with CRISPR/Cas9, allowing validation of sgRNA efficiency and Cas9 protein activity [23, 28, 29]. Currently, there is a tendency to develop DNA-free gene editing allowing specific changes in the genome and creating genetic modifications that are not GMO [30]. Precisely, the system to achieve these editing platforms uses in vitro assembled sgRNA/Cas9 ribonucleoprotein (RNP) complexes [31]. In plants, the entry of these complexes is done using protoplasts, where after a plant regeneration system, it allows obtaining transgene-free germplasm, which could represent advantages by allowing its rapid commercialization if it were a crop with commercial interest [32]. Genetic manipulation on protoplasts generally uses polyethylene glycol (PEG) due to simplicity because RNP complex is enclosed in PEG vesicles and fused with protoplasts, efficiency, low cost, and zero interference with the protoplast viability [33–36]. Therefore, recently, gene editing programs have increased interest in protoplast technology [19].

Few studies have reported protoplasts isolation in *R. communis*: from mesophyll cells [14, 37, 38], hypocotyls [39], endosperm [40], and calluses [14], describing moderated process efficiency and viability due to protoplast stresses, influencing the ability to regenerate cell wall and advance towards cellular division. Thus, further scientific knowledge regarding effective in vitro protoplast isolation with high viability for this promising species is necessary. Here, an efficient protoplast isolation and culture process in *R. communis* was induced*,* evaluating mesophyll and callus cells and a mix of enzymes, incubation times, and regeneration plant response. Furthermore, a PEG-mediated protoplast transfection was established to introduce DNA into *R. communis* genotypes cultivated in Colombia.

## Main text

### Materials and methods

#### Plant material

*R. communis*-VERC02 seeds were removed from the teste and cuticle in a laminar flow chamber, immersed in 70% ethanol 30 s, and washed with sterile water. Subsequently, the seeds were immersed in 0.1% Mercuric chloride (HgCl_2_) 3 min; finally, they were washed seven times with sterile water. Afterward, the seeds were placed in a Woody Plant Medium (WPM). Germinated seedlings were transferred to an autoclaved elongation medium in WPM [41] supplemented with 150 mg/L Casein, 50 mg/L Glutamine, 25 mg/L Adenine, 15 mg/L Arginine, 30 g/L Sucrose, 0.5 mg/L Cupric sulfate (CuSO_4_), 1.5 g/L Active charcoal and 2 mg/L Kinetin, pH 5.7. The seedling was in a natural photoperiod (12 h light, 12 h dark, 24 °C) and, every 2 weeks, sub-cultured [13]. From these seedlings grown under in vitro conditions (Plant Biotechnology Lab, EAFIT University), leaves were used for protoplast isolation.

#### Protoplasts isolation

15-day-old castor plants from in vitro conditions were left in 24 h dark photoperiod. Approximately 1 g of young leaves were collected and cut into thin strips (2 mm) using a sterile scalpel under laminar flow chamber conditions. These leaves were pre-plasmolyzed in 20 ml of Solution I [3 mM of Calcium chloride dihydrate (CaCl_2_∙H_2_O), 0.7 mM of Potassium dihydrogen phosphate (KH_2_PO_4_), 0.5 M mannitol, and 5 mM 2-(N-morpholino) ethanesulfonic acid (MES)], previously sterilized with a 0.22 µm filter (Sartorius, France), and shaken (45 rpm, 25 °C, 1 h). Leaf strips were transferred to an enzyme solution containing different enzymes (dissolved in Solution I) [pH 5.6] and exposure time (Table 1). The enzymes used in the treatments were: Cellulase *Aspergillus niger* (Sigma-Aldrich, USA), Pectinase *Aspergillus niger* (Sigma-Aldrich, USA), Hemicellulase *Aspergillus niger* (Sigma-Aldrich, USA), Peptinase *Rhizopus* sp. (Sigma-Aldrich, USA), Cellulase “*Onozuka*” R-10 (Yakult Pharmaceutical ind. Co. Ltd, Japan) and Macerozyme R-10 (Yakult Pharmaceutical ind. Co. Ltd, Japan). Enzyme concentrations and incubation time were chosen according to previous reporters [36, 42]. In treatment 4, only 3 incubation times were considered since the Macerozyme-R10 works as a multienzyme system with high catalytic activity by pectinase and hemicellulase [43].

**Table 1:** Enzyme concentration and incubation times evaluated for the protoplast isolation from mesophyll in *Ricinus communis*

Treatment	Enzymatic concentration (% w/v)	Incubation time (h)
T1	Cellulase 1%	8, 10, 12, 14, 16, 18
	Pectinase 1%	
	Hemicellulase 0.15%	
T2	Cellulase 2%	8, 10, 12, 14, 16, 18
	Pectinase 2%	
	Hemicellulase 0.3%	
T3	Cellulase 1.5%	8, 10, 12, 14, 16, 18
	Peptinase *Rhizopus* sp*.* 0.4%	
T4	Cellulase “Onozuka” R-10 1.6%	14, 16, 18
	Macerozyme R-10 0.8%	

The plant material was incubated with the enzyme solution in a shaker [dark at 29 °C 50 rpm]. Then, digested leaves were filtered through a 100 mesh (140 µm) sieve (Cell dissociation sieve-tissue grinder kit, Sigma-Aldrich, USA) previously sterilized (autoclave). The cells were centrifuged at 1000 rpm 5 min (Rotina 380 Hettich) and washed twice with 10 ml of plant protoplast digest/wash solution (Sigma-Aldrich, USA) to remove the enzyme solution. The purified protoplasts were counted using a Neubauer chamber (Bosco-Germany) and microscope (Zeiss Primo Star, Germany). The population density of the protoplasts (P) was calculated using the formula: X*10^4^ = protoplast/g FW (Fresh weight), where X is the average P number of the fields in the Neubauer chamber (0.100 mm). After that, the Trypan blue dye staining method (0.4%) was used to determine protoplast viability [14]. For each treatment, three replicates were performed. The results were evaluated using an ANOVA-one way with 95% confidence and the LSD multi-range test using STATGRAPHICS Centurion XVIII.

#### Protoplast culture

Protoplasts were initially cultured in WPM liquid without growth regulators, increased sucrose concentration to 60 g/L, and incubated in Erlenmeyer [dark at 25 °C 7 days]. Then, they were grown in a semi-solid medium [supplemented with 2 mg/L of Kinetin, 1.0 mg/L of 6-Benzylaminopurine (BAP), and 1.0 mg/L of Naphthaleneacetic acid (NAA)] in dark 45 days, seeking callus induction. For this procedure, a mixture of the protoplast suspension was made with the semi-solid medium [36ºC]. Then, the small calluses visualized were subcultured in the same medium.

#### PEG-mediated protoplast transfection

The pGH00.0126 plasmid (Addgene plasmid # 64257) was used for protoplast transfection. It includes a green fluorescent protein (EGFP) gene and a neomycin phosphotransferase II (*nptII*) marker gene, each one driven by the E12-Ω promoter, which is a strong constitutive promoter that contains the 50-upstream sequence of the cauliflower mosaic virus 35S promoter (CaMV) [44] (See Additional file 1). Approximately 1 × 10^5^ protoplasts were centrifuged [1500 rpm 3 min (Rotina 380)] and resuspended in 200 μl of MMG solution (0.4 M mannitol, 15 mM Magnesium chloride (MgCl2), 4 mM MES). Subsequently, 10 µg of plasmid was added and mixed gently by inversion. An equal volume of a freshly prepared solution of 40% (w/v) PEG solution with 0.2 M mannitol and 100 mM Calcium chloride (CaCl_2_) was added slowly, and the mixture was incubated [dark, room temperature, 15 min]. After the incubation, 950 µl of W5 solution (154 mM Sodium chloride (NaCl), 125 mM Calcium chloride (CaCl_2)_, and 5 mM Potassium chloride (KCl), 2 mM MES, 5 mM glucose) was added. The mixture was centrifuged [1500 rpm 3 min], and the supernatant was discarded. This last centrifugation was repeated once with 1 ml of W5 solution. Finally, the protoplast pellet was resuspended in 500 μl of growth medium (WPM, 30 g/L sucrose, 150 g/L citric acid, 150 g/L hydrolyzed casein, 0.5 mg/L CuSO4, 50 mg/L glutamine, 25 mg/L adenine, 15 mg/L arginine, and 20 ml/L coconut water), and incubated [dark, room temperature, 72 h].

#### Fluorescence signal detection and molecular evaluation of transfected protoplasts

Transfection efficiency was detected under fluorescence microscopy Zeiss Axio Scope.A1 with lamp HXP 120 V, and calculated as follows: transfection efficiency (%) = (bright green fluorescent protoplast number in view/total protoplast number in view) × 100% [36]. After 72 h of dark photoperiod, 200 μl of transfected protoplasts were collected by centrifugation [14.000 rpm 5 min], and genomic DNA was extracted [45]. Afterward, molecular characterization was carried out by amplifying the *nptII* gene found in the plasmid using nptII F (5′-TCA GTG GAA CGA AAA CTC ACG- 3′) and nptII R (5′-GCA AGG AAC AGT GAA TTG GAG T-3′) primers. The PCR conditions were 94 °C 4 min, 34 cycles at 94 °C 30 s, annealing at 52 °C 30 s, polymerization at 72 °C 30 s, followed by 72 °C 5 min. Finally, the PCR-amplified fragments were separated on a 1.2% agarose gel with 70 V 90 min.

#### Statistical analysis

We used a unifactorial design. The study factor was treatments, and each level was enzymatic concentrations in the digestion solutions and the digestion time. All experiments were replicated three times. The number of protoplasts obtained and viability percentage were considered response variables for the statistical analysis. All data were analyzed using analysis of variance (ANOVA-one way), and probability values of P ≤ 0.05 were considered significant. The difference among the treatment means was estimated using multiple ranges Least Significant Difference (LSD) test using the Statgraphics Centurion XVIII software.

## Results and discussion

### Effects of enzymatic composition and digestion time on protoplast isolation

**Fig. 1:**
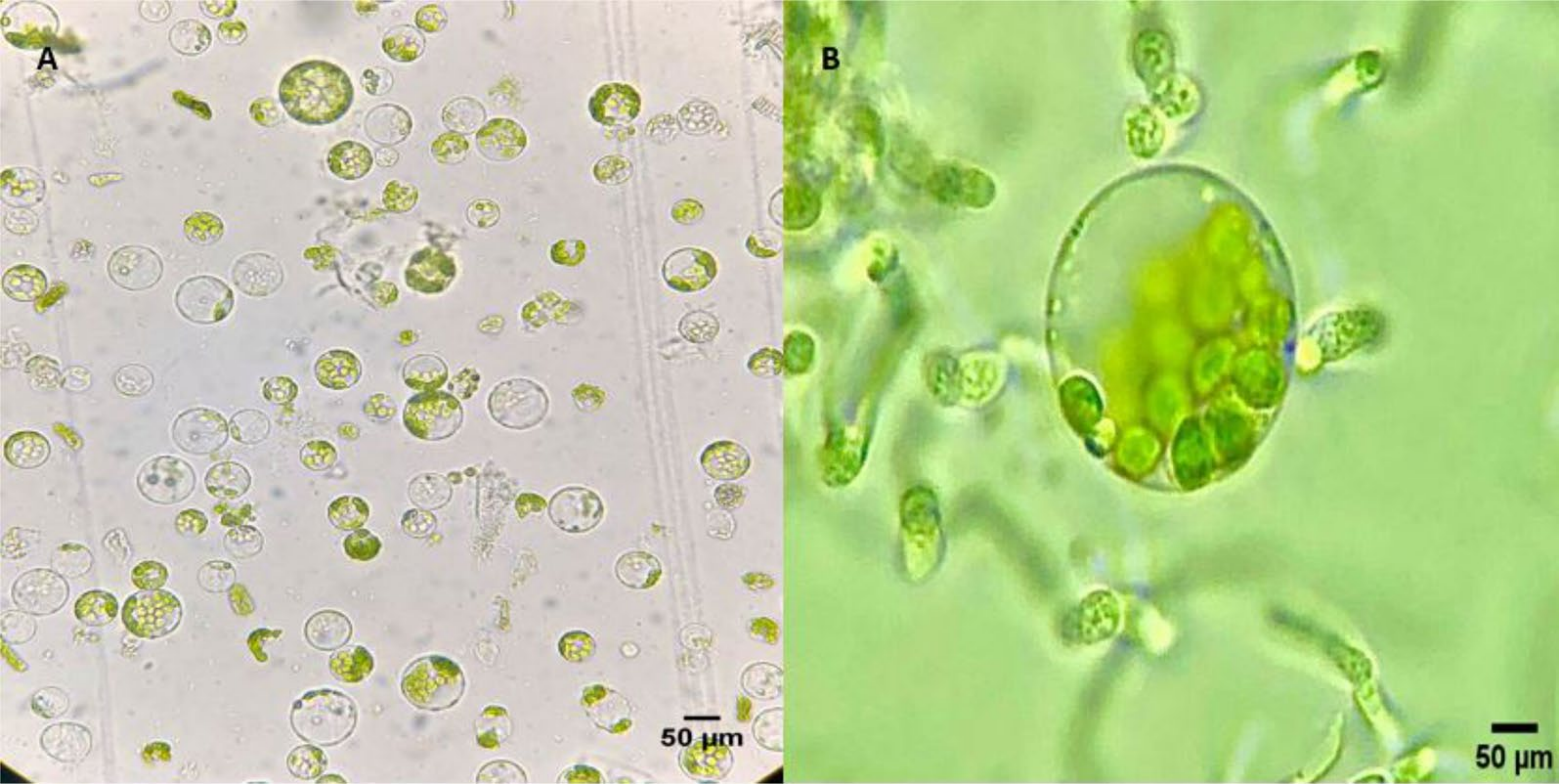
**A**, **B** Protoplast from *Ricinus communis* leaves obtained using enzyme treatment 4 (1.6% Cellulase Onozuka R-10 and 0.8% Macerozyme Onozuka R-10 with 16 h incubation at 29 °C, see Table 1) Objective 40x

**Fig. 2:**
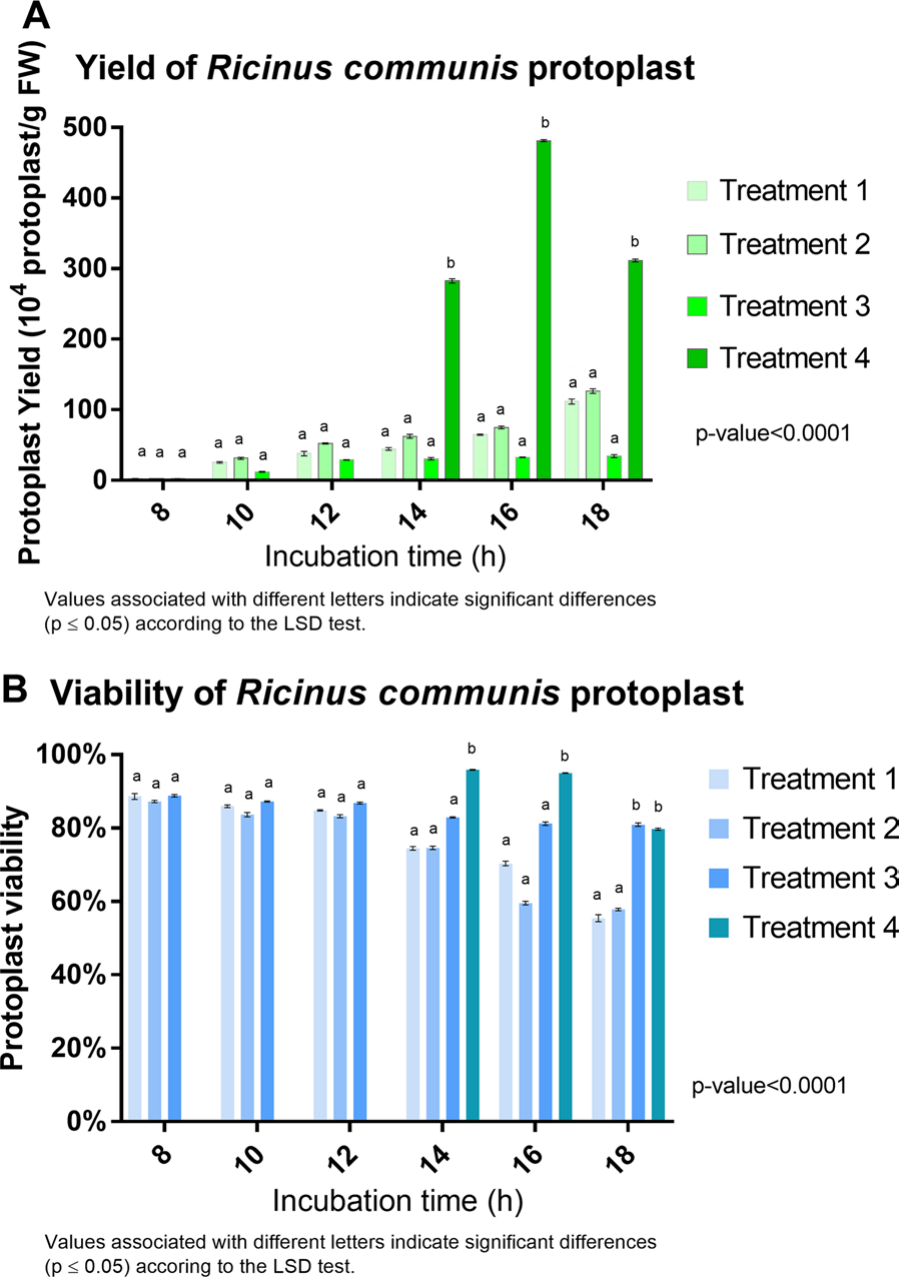
**A** Yield of protoplasts obtained from *Ricinus communis* leaves in different enzymolysis times. **B** Viability of *Ricinus communis* protoplast in different enzymolysis times. Values represent the mean ± SE of three experimental replications. The LSD test determined statistical significance. The same letters indicate no significant difference (P < 0.05)

**Fig. 3:**
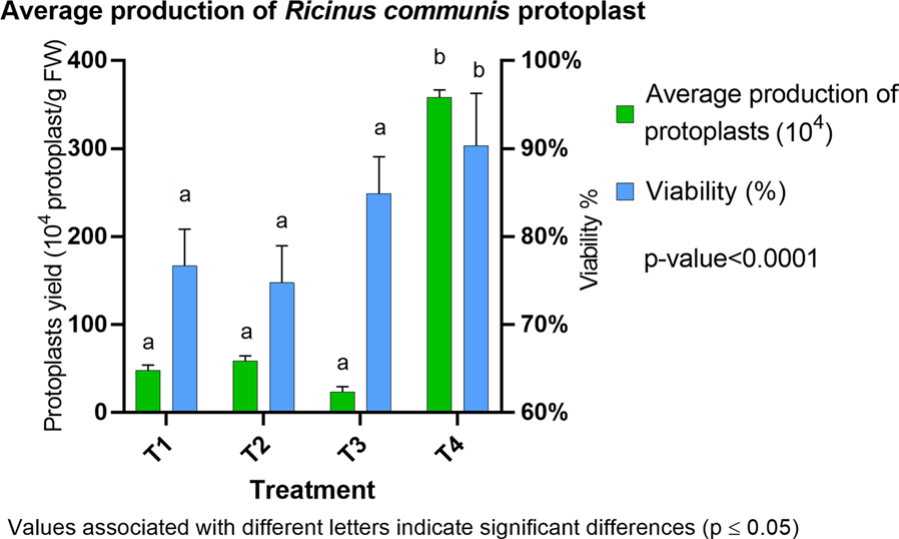
Average production of protoplasts obtained from the mesophyll of *Ricinus communis.* Values represent the mean ± SE of three experimental replications. The LSD test determined statistical significance. The same letters indicate no significant difference (P < 0.05)

The total protoplast yield varied, ranging from 2.1 to 481.16 × 10^4^ protoplasts/g FW. Treatment 4, with 1.6% Cellulase Onozuka R-10 and 0.8% Macerozyma Onozuka R-10, with 16 h of incubation, was the best condition to achieve a high yield of protoplasts (481.16 × 10^4^ protoplasts/g FW) with a high percentage of viability (95%) (Figs. 1 and  2). As displayed in Fig. 2, treatments 1, 2, and 3 have similar means of protoplast production and viability and differ from treatment 4 with a confidence level of 95% (p-value < 0.0001), here the best incubation time to produce protoplasts with greater viability and yield is in the range of 14–16 h. When protoplasts were incubated with the same solution for only 14 h, a higher viability percentage was achieved (96%) but with a lower protoplast concentration (282.5 × 10^4^). Figure 2a shows the protoplast/g FW obtained by each treatment listed in Table 1; similarly, production is contrasted with the viability (%) (Fig. 2b) during digestion time with enzyme concentrations. Figure 3 represents the results obtained after performing the LSD analysis; here, it is shown that the average protoplast yield/g FW was higher for treatment 4. Our results demonstrate a higher yield of *R. communis* protoplasts obtained per gram of fresh leaf tissue and noted viability compared with other reports. [14, 37] obtained protoplast concentrations of 8.5 × 10^5^ and 1.19 × 10^6^, respectively, in the variety evaluated, demonstrating response genotype-dependent. Similarly, [46] reported a maximum of 1.18 × 10^6^ protoplast/g FW with viability of 76.03%. This shows that the concentrations, incubation time, and osmotic solutions reported in this article achieve high concentrations of protoplasts with good levels of viability concerning the most recent reports of isolation of mesophyll protoplasts from castor bean leaves. On the other hand, we used a similar concentration (2% cellulase Onozuka R10 and 2% Pectinase) reported by [14] to obtain protoplasts from callus. We achieved cells elongated and parenchymal after the enzymatic process (in callus tissue), which does not coincide with those previously published by [14], who described protoplasts obtained from calluses as colorless, spherical, and larger than mesophilic. Although the enzyme concentration was gradually increased (and hemicellulase was even added), protoplast isolation was impossible. Instead, the viability of the cells decreased every time enzyme concentration increased, demonstrated with the trypan blue dye staining method. However, the concentration of the proposed osmotic stabilizer was adequate for the cells to be plasmolyzed.

### Protoplast culture of *R. communis*

**Fig. 4:**
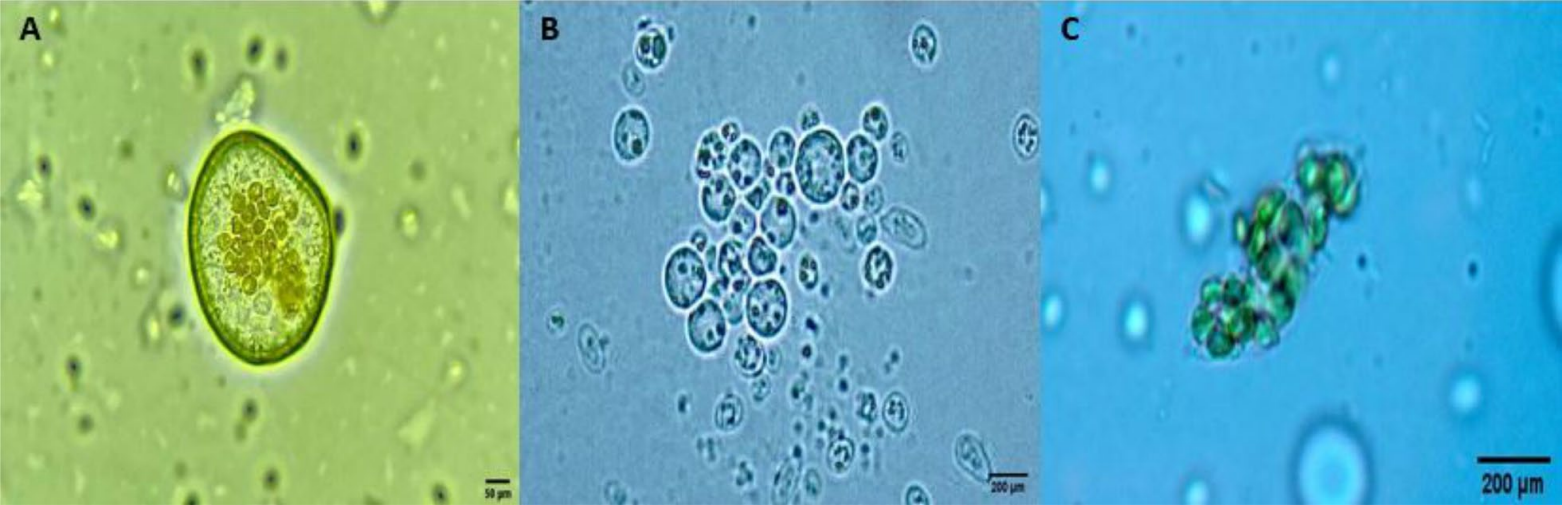
**A**–**C**. Process of microcolony formation from *Ricinus communis* protoplasts. **A**, **B** Small colonies of *Ricinus communis* microcallus formed from protoplast culture. **C** Microcolonies formed 20 days after isolation. Objective 100x

Isolated protoplasts were transferred to the suspension culture medium (WPM supplemented with hormones) for microcallus formation (Fig. 4). These microcallus colonies were obtained after 8 weeks of culture, with sub-cultures every 15 days. A protoplast culture system's success mainly consists of consistently producing a large population of uniform protoplasts with high viability [47, 48]. In our case, a high number of viable protoplasts was obtained. However, few colonies were managed. Indeed, the newly isolated protoplasts require osmotic stabilization by adding high glucose or mannitol concentrations. However, when we added a high glucose concentration (60 g/L), cells remained stable, as protoplasts, without regeneration to form organogenic calli. Despite this, in this study was possible to establish an initial medium for protoplast formation to microcolonies that were formed in microcalli in 8 weeks, with stable concentrations of Kinetin (2 mg/L), BAP (1 mg/L), and NAA (1 mg/L). The success of protoplast isolation to shoot regeneration, essential for a biotech-based platform, remains as a bottleneck for many plant species due to several factors [22]; among them, tissue, culture medium, and environmental factors that influence protoplast and protoplast-derived cell's ability to express their totipotence and growth. Because castor bean is recalcitrant, reports on protoplast isolation [14, 37–39] are only successful until callus formation, without plant regeneration, as in our case. Excellent yield, quality, and high viability of protoplasts derived from mesophyll cells are essential factors achieved in this research and facilitate future genetic transfection, metabolite production, and molecular breeding in *R. communis*.

### Establishment of a PEG-mediated protoplast transfection in *R. communis*

**Fig. 5:**
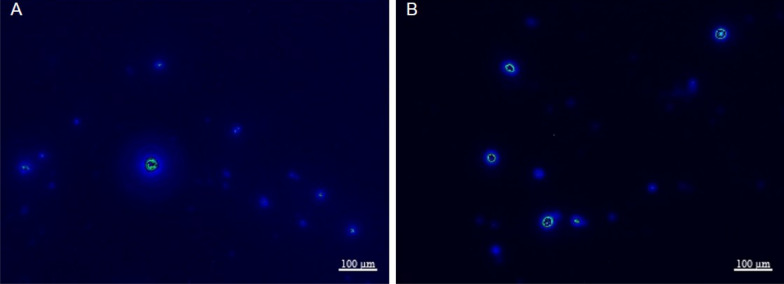
Detection of GFP fluorescence in transformed *Ricinus communis* mesophyll protoplasts with pGH00.0126 plasmid by fluorescence microscope. **A**, **B** show protoplasts in the fluorescence microscope fields expressing the GFP reporter gene. Objective 40x

After the PEG-mediated transfection process, a transfection efficiency of 50.7% was observed, a higher efficiency than reported by [29], which was 12.37%. However our results were similar to those obtained by [49], where a 51% transfection efficiency was reported. GFP signal was visible in protoplasts transfected with 10 µg of plasmid (Fig. 5). In the same way, we determined the presence of the *nptII* gene, amplifying PCR-specific products, not present in the negative control. This molecular evidence, added to the detection of the GFP signal, demonstrates the success of an efficient transfection system in castor bean protoplasts (Fig. 5 and Additional file 2). Here it was possible to establish a simple procedure to introduce genetic material like plasmid DNA. The above presents a helpful tool for genetically improving castor bean programs since our protocol can eventually be replicated using genetic editing tools such as CRISPR/Cas9 through ribonucleoproteins entry protoplasts, and in this way, obtain experimental systems of a single cell.

In conclusion, we describe a simple, efficient, and replicable method for protoplast-mediated production of transformed *R. communis* lines based on PEG-mediated protocol. It was limited when pretending to isolate them from castor bean callus in the evaluated variety. We found that the combination and concentration of the enzymes had a significant effect on the protoplast isolation because it provides obtaining *R. communis* protoplasts with high levels of viability that was subsequently reflected in the formation of microcolonies of microcallus. Furthermore, our performed assay showed significant results regarding a high concentration of protoplasts (481.16 × 10^4^ protoplasts/g FW) obtained and a high viability percentage (95%). In addition, it was possible to carry out the PEG-mediated transformation of protoplasts of a plasmid with the GFP reporter gene, reaching good percentages of transfection efficiency (50.7%). Moreover, it was possible to establish a protoplasts-derived system to regenerate microcallus in *R. communis*. Nonetheless, further research is required to optimize the plant regeneration system and link the single-cell isolation technique until the regeneration of the plant. Our results support scientific bases to develop gene-editing studies with CRISPR/Cas9 plasmids or ribonucleoproteins since modifying protoplasts is a susceptible and convenient method to evaluate the generated editing rates and thus have a whole system to obtain DNA-free cultures. All this expands the range of applications at the level of genetic improvement of *R. communis* varieties.

## Limitations

Further research is needed to optimize the plant regeneration system and link the single-cell isolation technique until the regeneration of the plant concerning the recalcitrance of this species.

## Supplementary Information

**Additional file 1: **Overview of the plasmid used in transfection of *R. communis* protoplasts.

**Additional file 2: **Agarose gel electrophoresis showing amplified PCR-products from the *nptII* gene to confirm the transformation of *R. communis *protoplast with the pGH00.0126 vector. This molecular characterization was made using transfected protoplasts after 72 hours of incubation.

## Data Availability

The datasets used and/or analyzed during the current study are available from the corresponding author upon reasonable request.

## Abbreviations

HgCl_2_: Mercuric chloride
FW: Fresh weight
WPM: Woody plant medium
CuSO4: Cupric sulfate
CaCl2∙H2O: Calcium chloride dihydrate
KH2PO4: Potassium dihydrogen phosphate
MES: 2-(N-morpholino) ethanesulfonic acid
BAP: 6-Benzylaminopurine
NAA: Naphthaleneacetic acid
EGFP: Green fluorescent protein
*nptII*: Neomycin phosphotransferase
CaMV: Cauliflower mosaic virus 35S promoter
MgCl2: Magnesium chloride
CaCl2: Calcium chloride
NaCl: Sodium chloride
KCl: Potassium chloride
LSD: Least significant difference
