# Supplementary material for: An efficient method for protoplast-mediated production of transformed castor bean (Ricinus communis) lines
Source: BMC Res Notes. 2023 Jul 6;16:140. doi: 10.1186/s13104-023-06414-y (PMC10327310; doi:10.1186/s13104-023-06414-y)
Supplement: Supplementary file 1 — Additional file 1: Overview of the plasmid used in transfection of R. communis protoplasts. [file 13104_2023_6414_MOESM1_ESM.docx]

**Supplementary Files**

Additional file 1. Overview of the plasmid used in transfection of *R. communis* protoplasts.


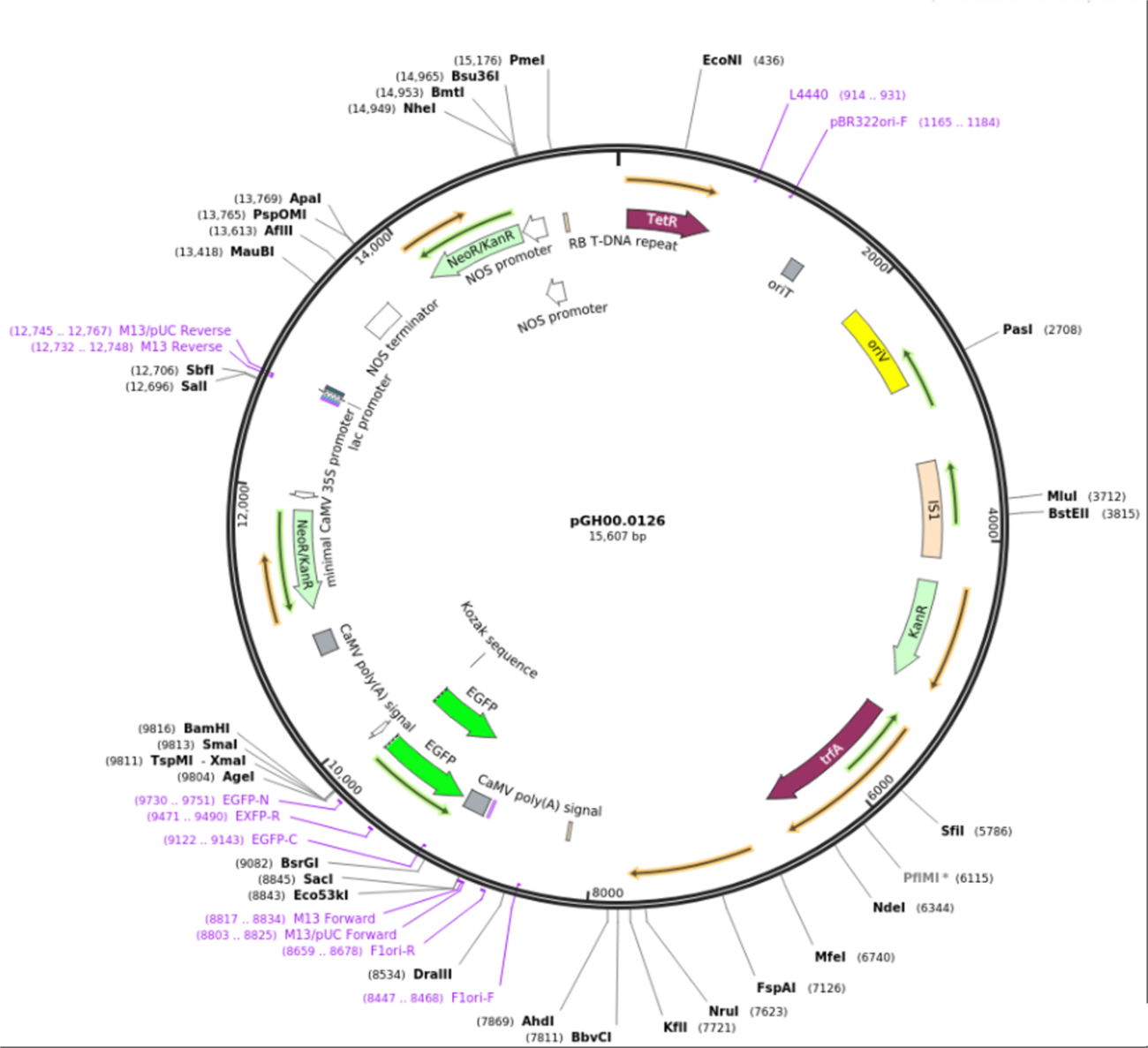


The 15,607 bp-long plasmid: the EGFP gene under the E12-omega promotor; the neomycin phosphotransferase II gene under E12-omega promotor; the promoter consists of two repeated copies of the cauliflower mosaic virus (CaMV) 35S promoter, followed by one copy of the P35S.
