# Supplementary material for: An efficient method for protoplast-mediated production of transformed castor bean (Ricinus communis) lines
Source: BMC Res Notes. 2023 Jul 6;16:140. doi: 10.1186/s13104-023-06414-y (PMC10327310; doi:10.1186/s13104-023-06414-y)
Supplement: Supplementary file 2 — Additional file 2: Agarose gel electrophoresis showing amplified PCR-products from the nptII gene to confirm the transformation of R. communis protoplast with the pGH00.0126 vector. This molecular characterization was made using transfected protoplasts after 72 hours of incubation. [file 13104_2023_6414_MOESM2_ESM.docx]

Additional file 2. Agarose gel electrophoresis showing amplified PCR-products from the *nptII* gene to confirm the transformation of *R. communis* protoplast with the pGH00.0126 vector. This molecular characterization was made using transfected protoplasts after 72 hours of incubation.


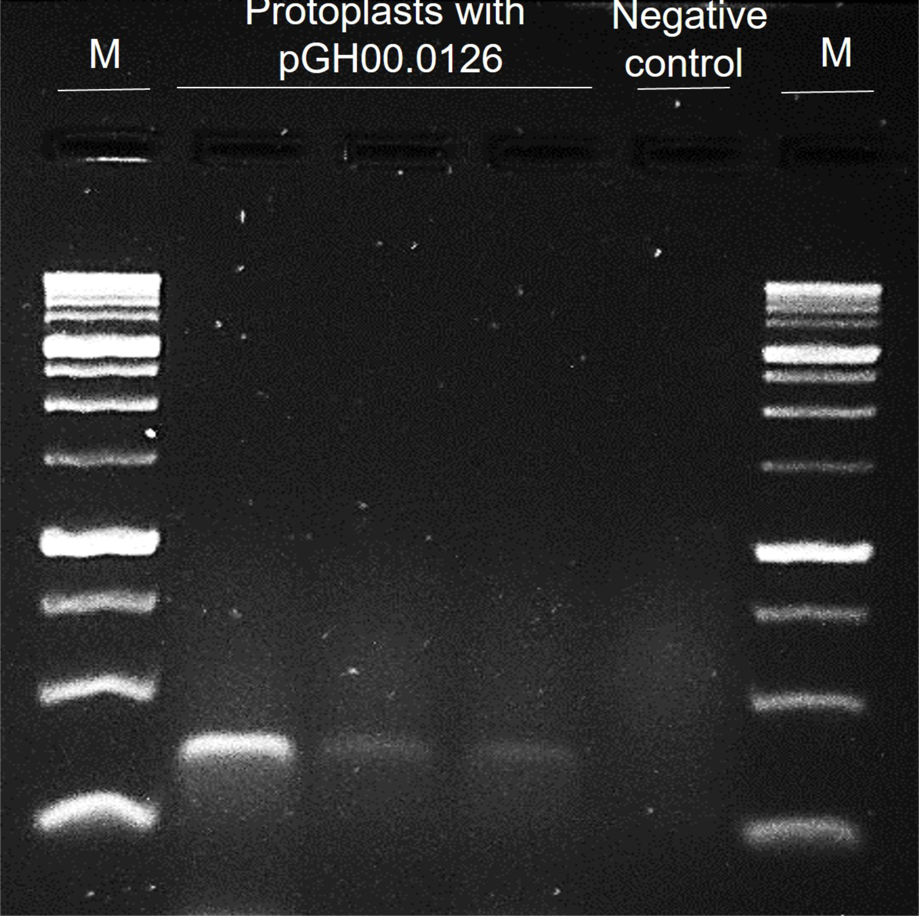


M: molecular weight marker; Transfected protoplasts with pGH00.0126 vector; Negative control: non-transfected protoplasts. PCR-products correspond to *nptII* gene fragments in transfected protoplasts, after 72 hours of incubation.
